# Functionality of the human antibody response to *Candida albicans*

**DOI:** 10.1080/21505594.2021.2015116

**Published:** 2021-12-19

**Authors:** Melissa Wich, Stephanie Greim, Marta Ferreira-Gomes, Thomas Krüger, Olaf Kniemeyer, Axel A. Brakhage, Ilse D. Jacobsen, Bernhard Hube, Berit Jungnickel

**Affiliations:** a Institute of Biochemistry and Biophysics, Faculty of Biological Sciences, Friedrich Schiller University, Jena, Germany; b Leibniz Institute for Natural Product Research and Infection Biology, Hans Knöll Institute, Jena, Germany; c Institute of Microbiology, Faculty of Biological Sciences, Friedrich Schiller University, Jena, Germany

**Keywords:** Candida albicans, B cells, antibodies, human, neutralization, adhesion, monocytes, secreted aspartic proteinases

## Abstract

*Candida albicans* is a common commensal on human mucosal surfaces, but can become pathogenic, e.g. if the host is immunocompromised. While neutrophils, macrophages and T cells are regarded as major players in the defense against pathogenic *C. albicans*, the role of B cells and the protective function of their antibodies are less well characterized. In this study, we show that human serum antibodies are able to enhance the association of human THP-1 monocyte-like cells with *C. albicans* cells. Human serum antibodies are also capable of inhibiting the adherence and damage dealt to epithelial cells. Furthermore, human serum antibodies impair *C. albicans* invasion of human oral epithelial cells by blocking induced endocytosis and consequently host cell damage. While aspartic proteases secreted by *C. albicans* are able to cleave human IgG, this process does not appear to affect the protective function of human antibodies. Thus, humans are equipped with a robust antibody response to *C. albicans*, which can enhance antifungal activities and prevent fungal-mediated epithelial damage.

## Introduction

*Candida albicans* is a member of the human microbiota that colonizes mucosal surfaces of about 70% of healthy individuals [1]. Under certain circumstances, however, *C. albicans* can cause infections. These range from relatively benign superficial mycoses of the mucosae (oropharyngeal and vulvovaginal candidiasis) to life-threatening disseminated infections. Risk factors for the latter are prolonged treatment with antibiotics, disturbance of mucosal barrier function, and immunosuppression [2]. Due to problems with diagnosis and treatment of *C. albicans* infection, such invasive mycoses are associated with a high mortality rate [3,4]. Therefore, preventive treatment options for at risk patients are highly desirable.

The acute immune response to *C. albicans* infection is dominated by the innate immune system. Neutrophils constitute a first line of defense, carrying out the fungal clearance by phagocytosis [5]. Monocytes and macrophages may fulfil similar functions [6]. Among the cells of the adaptive immune system, T-helper 17 and also T-helper 1 cells have been found to aid in the fungal clearance by regulating the function of phagocytic cells [2,7]. While a role of B cell responses for antifungal defense was not evident in early studies, more recent work in murine models by Doron *et al*. showed that B cells contribute to protective responses in disseminated candidiasis [8]. The authors attributed the protective effect to induction of IgG, which is in line with antibody-mediated protection observed by De Bernardis *et al*. in experimental vaginal candidiasis [9]. However, antibody-independent protective effects of B cells by interaction with T cells were also suggested [10].

The antibody response to *C. albicans* can in fact serve as a diagnostic marker, as antibodies directed against fungal cell wall components increase upon fungal infection and may be easily quantified by ELISA. Also, antibodies against *C. albicans* proteins are detectable during infection [11], and the pattern of target recognition changes during the course of the infection, indicating an ongoing response of the B cells [12]. Vaccination approaches have been undertaken, and an antibody response to Als3, a major adhesin and invasin of *C. albicans*, was found to be protective in a model of vulvovaginal candidiasis [13].

In the context of immunosuppression, a protective antibody response has the advantage to act independently of other (potentially impaired) immune cells, such as neutrophils or macrophages. In order to do so, these antibodies need to be functional in the context of affecting virulence attributes of the pathogen, rather than functioning in opsonization or complement activation, which would require phagocytic activities for final clearance of the pathogen. Even though the studies mentioned above indicate protective functions of antibodies, the mechanisms by which protection is conferred have not been addressed in detail. Classical opsonization by cloned IgGs from humans promoted phagocytosis by macrophages [14]. Furthermore, De Bernardis *et al*. showed that antibodies can interfere with fungal adhesion to vaginal epithelial cells [9]. Whether similar mechanisms protect other types or epithelial cells is, however, not yet known.

Therefore, we elucidated the protective potential of human antibodies and investigated the underlying mechanisms. We show that human anti-*Candida albicans* antibodies can block *C. albicans* adherence to, invasion into and damage of oral epithelial cells. While IgG antibodies may be cleaved by aspartic proteases secreted by *C. albicans*, this does not interfere with their capacity to protect oral epithelial cells.

## Results

**Figure 1.**
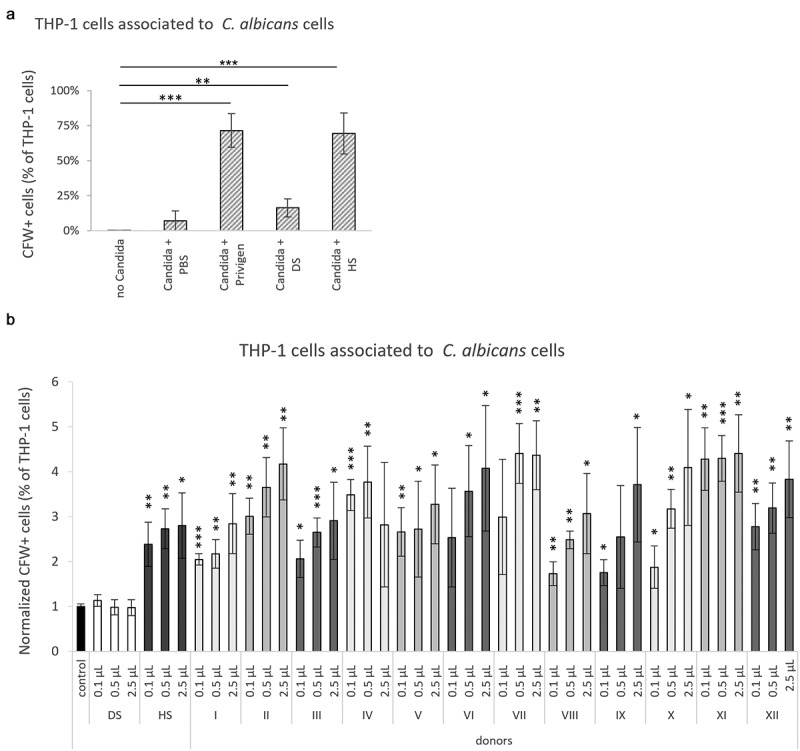
**Human antibodies enhance the interaction of THP-1 monocyte-like cells with *C. albicans* cells**. Heat-killed *C. albicans* yeasts were treated with human serum or antibodies for 30 min and stained with Calcofluor White (CFW). The interaction of THP-1 monocyte-like cells with *C. albicans* yeasts was determined by measuring the percentage of CFW-positive (CFW+) cells among the THP-1 cells by flow cytometry. (a) Treatment of heat-killed *C. albicans* yeasts with human serum (HS) and Privigen, but not antibody-depleted serum (DS) prior to co-incubation increases interaction with THP-1 cells. Data represent mean values ± SD of three independent experiments. (b) Interaction of THP-1 monocyte-like cells with heat-killed *C. albicans* yeasts treated with serum from 12 independent healthy donors. Results were normalized to the control sample (THP-1 cellco-incubation with *C. albicans* yeast that were treated with PBS instead of serum). Data represent mean values ± SD of three independent experiments. Statistical significances were calculated by comparing the samples to the PBS treated control with unpaired two-tailed t-tests. *p < 0.05, **p < 0.01, ***p < 0.001

To test the functional capacities of the human antibody response against *C. albicans*, we investigated the properties of commercially available human serum pooled from healthy donors and Privigen, a human IgG preparation isolated from pooled serum of donors, which is also used in therapeutic settings [15,16]. Privigen and human serum enhanced the association of human THP-1 monocyte-like cells with *C. albicans* cells, indicative of opsonization of *C. albicans*, while antibody-depleted serum did not (Figure 1(a) and Figure S1). Analysis of serum from 12 different healthy donors revealed dose-dependent opsonizing capacity in all of them (Figure 1(b)). Accordingly, antibodies recognizing *C. albicans* are present in human serum as well as in the Privigen preparation. Also, these antibodies showed the same opsonizing capacity as was observed for cloned isolated IgGs and human macrophages [14].

**Figure 2.**
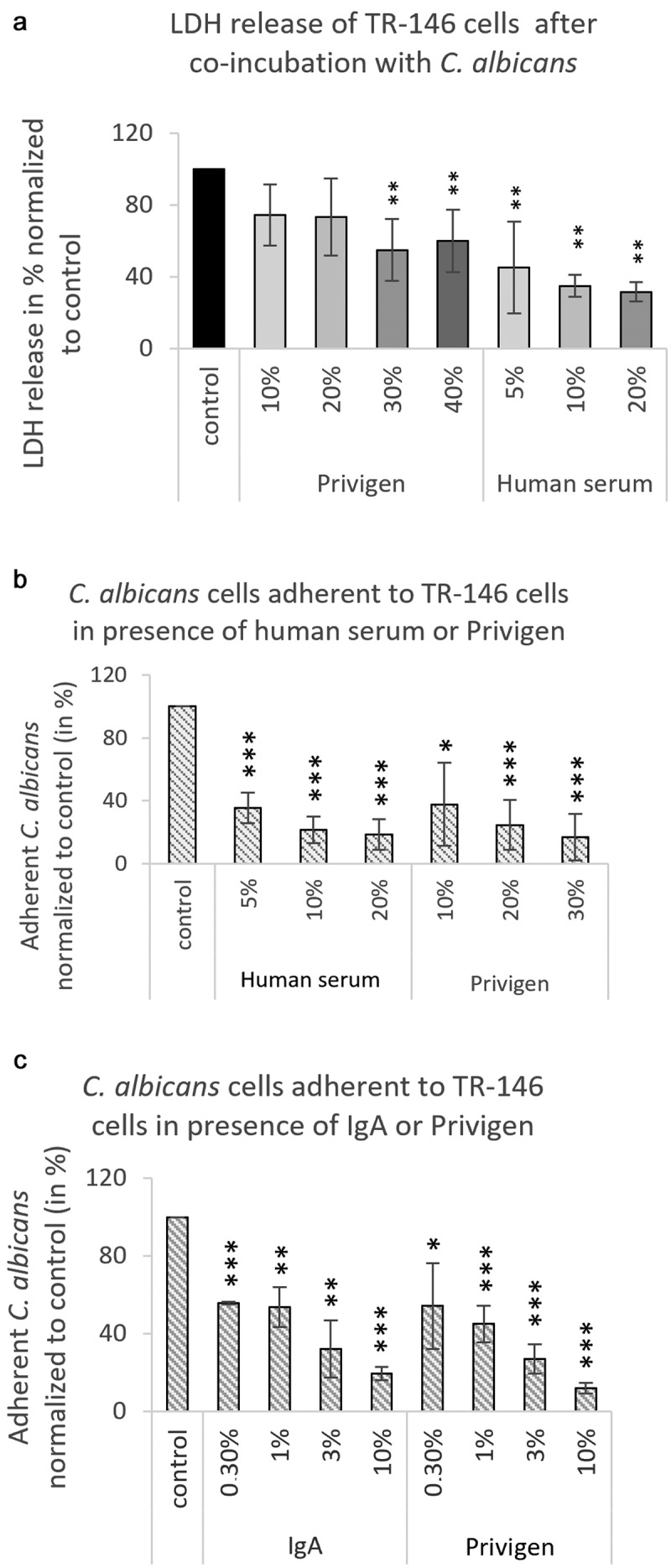
**Human antibodies decrease the damage dealt to oral epithelium cells by *C. albicans* cells and inhibit the adherence of *C. albicans* cells to TR-146 cells**. (a) *C. albicans* cells were co-incubated with TR-146 cells in 1 mL of cell culture medium containing varying concentrations of Privigen or human serum. Damage was assessed by measuring LDH release after 24 h of co-incubation. Data were normalized to the control samples and represent mean values ± SD of three independent experiments. (b-c) *C. albicans* cells were co-incubated with TR-146 cell grown on coverslips for 30 min in 1 mL culture medium containing varying concentrations of human serum (b), Privigen (b, c) or IgA (c). Adherent *C. albicans* cells were counted on 10 pictures taken with ten-fold magnification from each coverslip. Data were normalized to the control samples without serum or antibodies and represent mean values ± SD of four (B) or three (C) independent experiments. Statistical significances were calculated by comparing the samples to controls without serum or antibodies with unpaired two-tailed t-tests. *p < 0.05, **p < 0.01, ***p < 0.001

In addition to modulating or enhancing the activity of immune cells such as phagocytes, antibodies may interfere with the pathogenicity of microbes. Such neutralizing capacity is particularly important in settings of immunosuppression. In a first approach, we assessed the ability of antibodies to interfere with tissue damage by *C. albicans* in an *in vitro* infection model employing human oral epithelial cells (TR-146). Human serum and Privigen led to a significant dose-dependent decrease in epithelial damage, quantified as epithelial lactate dehydrogenase (LDH) release, caused by *C. albicans* (Figure 2(a)), implying that antibodies protecting from the damaging functions of *C. albicans* were also contained in the preparation. We conclude that the human antibody response to *C. albicans* can block fungal activities required to cause epithelial damage, which may be relevant in therapeutic applications.

Epithelial cell damage caused by *C. albicans* relies on fungal adherence and subsequent invasion. Invasion is mediated by two routes: induced endocytosis of epithelial cells triggered by fungal invasins, and active penetration of the fungus into epithelial cells [17]. To assess how antibodies can block *C. albicans*-induced epithelial damage, we investigated whether they inhibit either adherence or invasion as prerequisites for epithelial damage.

**Figure 3.**
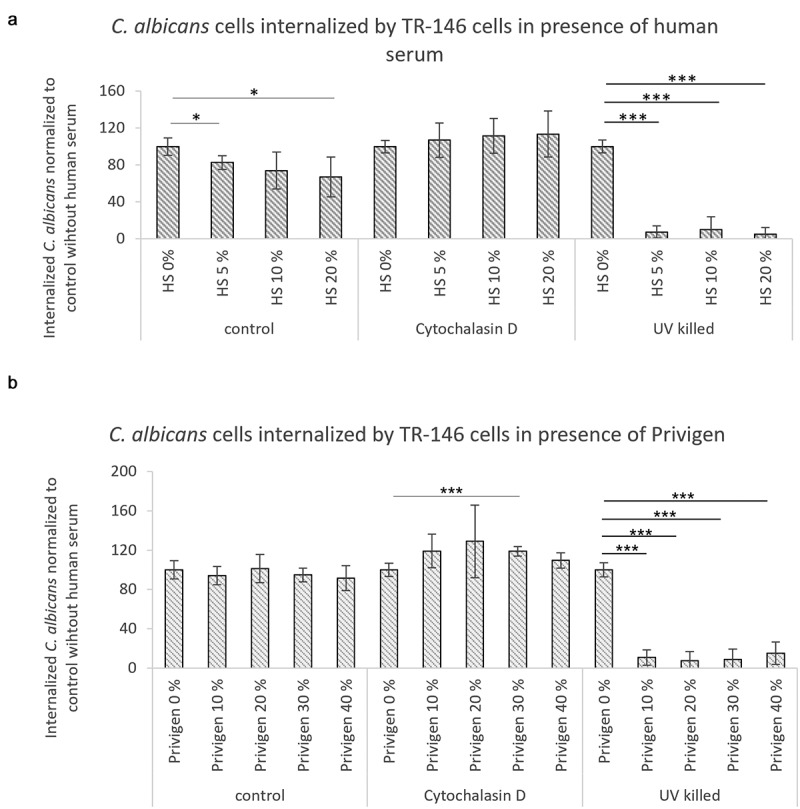
**Antibodies from human serum and Privigen reduce invasion via endocytosis of *C. albicans* by oral epithelial cells**. Invasion of *C. albicans* cells into TR-146 cells after co-incubation for 3 h in the presence of different concentrations of serum or Privigen was determined using fluorescence microscopy. A and B) Internalization of *C. albicans* cells (UV-killed or living) by TR-146 cells in the presence of Cytochalasin D and human serum (HS) (a) or Privigen (b). Data are normalized to the control samples without human serum or Privigen and represent mean values ± SD of the technical duplicates from two independent experiments. Statistical significances were calculated in comparison to the control sample using unpaired two-tailed t-tests. *p < 0.05, **p < 0.01, ***p < 0.001

Adherence of *C. albicans* cells to TR-146 cells was profoundly inhibited by both human serum and the Privigen preparation (Figure 2(b)). To determine if the neutralizing capacity is limited to the IgG isotype, we tested a human IgA preparation, the main antibody subtype at epithelial surfaces, isolated from colostrum. This preparation also reduced adherence of *C. albicans* to oral epithelial cells in a dose-dependent manner and to a similar extend as Privigen (Figure 2(c)), consistent with recent findings [18]. Privigen also inhibited the adherence of *C. albicans* to endothelial HUVEC cells (Figure S2A). Following adherence, subsequent invasion occurs either by induced endocytosis or active penetration [17]. We assessed the effect of human antibodies on induced endocytosis using UV-killed *C. albicans* cells (which cannot actively penetrate cells), and the effect on active penetration by inhibiting induced endocytosis via Cytochalasin D treatment (which inhibits actin polymerization and hence endocytosis). Interestingly, human antibodies inhibited induced endocytosis very efficiently, but were ineffective against active penetration of the host cells by the fungus (Figure 3). We thus conclude that human antibodies reduce tissue damage by *C. albicans* by interfering with both adherence and invasion via induced endocytosis.

**Figure 4.**
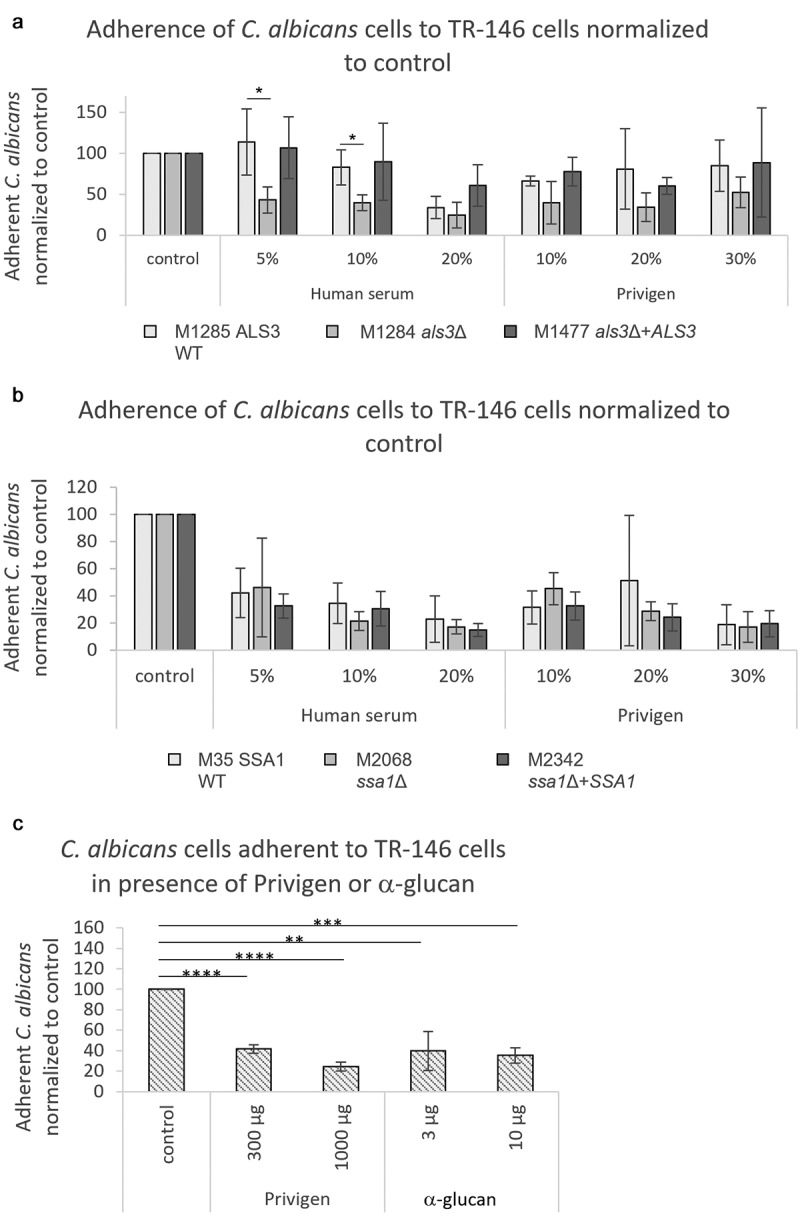
**Antibody-mediated inhibition of *C. albicans* adherence to oral epithelial cells does not depend on the *C. albicans* adhesins Als3 and Ssa1**. Adherence of *C. albicans* cells to TR-146 cells in the presence of different concentrations of serum or Privigen was determined by counting fungal cells adherent to fully confluent TR-146 cells on 10 pictures that were taken throughout the coverslip for each sample using fluorescence microscopy. (a-b) Adherence of wildtype (M1285 WT), ALS3 knockout (M1284 *als3*Δ) and ALS3 revertant (M1477 *als3*Δ +*ALS*3) strains (a) or the wildtype (M35 WT), SSA1 knockout (M2068 *ssa1*Δ) and revertant (M2342 *ssa1*Δ+*SSA1*) strains (b) to TR-146 cells after 1 hour of co-culture in the presence of different concentrations of human serum or Privigen. (c) Adherence of wildtype (SC5314) *C. albicans* cells to TR-146 cells after 30 min of co-culture in presence of different concentrations of Privigen or anti-fungal β-glucan. The number of adherent *C. albicans* is normalized to the number of adherent *C. albicans* in the control sample of the respective strain that does not contain serum or Privigen. Data represent mean values ± SD of three independent experiments. Statistical significances were calculated using unpaired two-tailed t-tests. *p < 0.05, **p < 0.01, ***p < 0.001

Adherence and induced endocytosis both involve the *C. albicans* protein Als3, which is a promising vaccine target [19,20]. Since serum and Privigen interfered with both processes, we hypothesized that the protective effect is mediated by antibodies targeting Als3. To test this, we used a *C. albicans* Als3 deletion mutant. Surprisingly, the effect of Privigen on adherence was more pronounced for the *C. albicans* Als3 deletion mutant (Figure 4(a) and Figure S2B) than for the wildtype and revertant strain. This implies that non-Als3 adhesins were targeted by the functional anti-*Candida* antibodies contained in Privigen. Another prominent *C. albicans* adhesin, Ssa1, was also excluded as single antibody target by using a corresponding deletion mutant (Figure 4(b) and Figure S2C). To test whether antibodies against cell wall carbohydrates affect adhesion of *C. albicans*, we directly tested the effect of anti-beta glucan antibodies on adhesion and detected a clear inhibitory capacity (Figure 4(c)). These results imply that naturally occurring anti-*Candida* antibodies act by targeting several factors involved in adherence or by impairing general surface adherence properties such as hydrophobicity.

**Figure 5.**
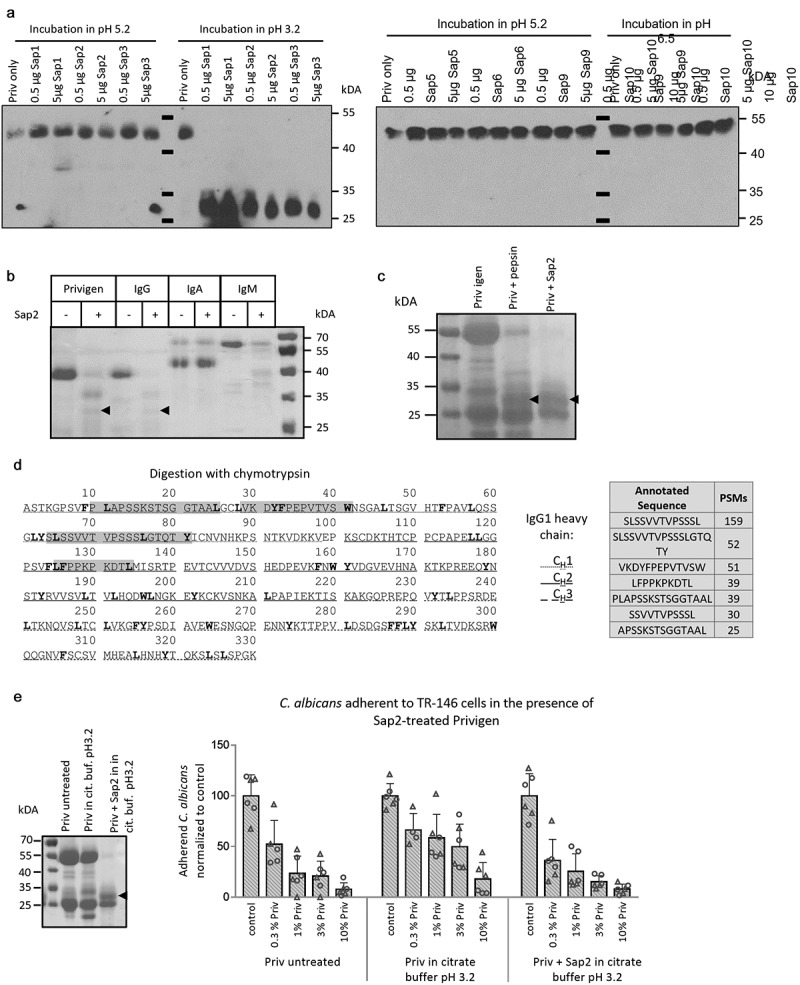
**The secreted *C. albicans* proteinases Sap1, Sap2 and Sap3 cleave human antibodies, which retain their inhibitive function in adherence assays**. (a) Privigen was incubated with different Saps in citrate buffer with either pH 5.2, or the pH optimum matching the respective Sap (pH 3.2 for Sap1, Sap2 and Sap3; pH 5.2 for Sap5 and Sap6; pH6.5 for Sap9 and Sap10). Western blot detection using anti-human-IgG antibodies show the 50 kDa heavy chain of IgG antibodies and a ~ 30 kDa fragment appearing after Sap incubation. The pictures shown are representative of three independent experiments. (b) Human IgM, IgA, IgG and Privigen were incubated with Sap2 in citrate buffer with pH 3.2. The ~30 kDa fragment of cleaved IgG is highlighted with arrows. The pictures shown are representative of three independent experiments. (c) Privigen (Priv.) was incubated with Sap2 or pepsin in citrate buffer with pH 3.2. Samples containing 15 µg Privigen were prepared for SDS-PAGE and Western blotting using anti-human-IgG antibodies for detection. The ~30 kDa fragment of cleaved IgG is highlighted with arrows. The pictures shown are representative of two independent experiments. (d) The peptide sequence of the constant region of the human IgG1 heavy chain (uniprot-ID: P01857) is depicted. Fragments of ~30 kDa in samples of Sap2 treated Privigen were excised from the Coomassie-stained polyacrylamide gel and prepared for LC-MS analysis following chymotryptic digestion (cleavage sites are highlighted by bold letters in the sequence). The annotated sequences of peptides identified by LC-MS are marked in gray in the sequence. The abundance of each of the identified peptides is represented by the PSM value (peptide spectrum matches). (e) Left: Representative SDS-Page gel picture showing the successful cleavage of the IgG heavy chain, the ~30 kDa fragment of cleaved IgG is highlighted with an arrow. Right: Adherence of *C. albicans* cells to TR-146 cells in the presence of Privigen that had been incubated in citrate buffer pH3.2 with Sap2 was determined by counting fungal cells adherent to fully confluent TR-146 cells on 10 pictures that were taken throughout the coverslip for each sample using fluorescence microscopy. Data represent the values of two independent experiment (circles and triangles) ± SD

Proteins from *C. albicans* culture supernatant have been shown to cleave human antibodies as a potential immune escape mechanism [21], and early reports suggest an involvement of secreted aspartic proteases (Saps) of *C. albicans* in this phenomenon [22–24]. We therefore tested different Saps for their ability to cleave, and thereby potentially inactivate, the IgG antibodies contained in Privigen. Interestingly, Sap1, 2 and 3 were indeed able to cleave IgG when employed at their pH optimum (Figure 5(a), left panel), while Sap5, 6, 9 and 10 appeared unable to do so (Figure 5(a), right panel). While IgG and IgM were efficiently cleaved by Sap2, IgA was apparently protected from cleavage (Figure 5(b)). The pattern of IgG cleavage strongly resembled the one generated when IgG was incubated with pepsin, which generates F(ab)_2_ fragments (Figure 5(c)). Indeed, mass spectrometric analysis of the fragment(s) (Figure S3) revealed sequences from Ig variable regions and the CH1 domain, suggesting that the Fab or F(ab)_2_ fragments stayed intact upon antibody cleavage by Sap2 (Figure 5(d)). Therefore, the cleaved IgG antibodies might maintain some functionality upon cleavage by Saps. As this might be relevant in a therapeutic setting, we tested the consequences of antibody cleavage by Sap2 on *C. albicans* adherence properties. Privigen IgG was cleaved completely with Sap2 (Figure 5(e), left panel) but the cleaved antibodies inhibited adherence of *C. albicans* cells to human cells as efficiently as the non-cleaved Privigen preparation (Figure 5(e), right panel). Thus, even though Saps can cleave IgG antibodies, they retain their functionality in preventing *C. albicans* adherence.

## Discussion

Recent studies have indicated a protective role of B cells and antibodies in defense against systemic candidiasis [8,10,14,25]. Antibodies can enhance antifungal activity of phagocytes by opsonization [26]. Here, however, we demonstrate that naturally occurring human antibodies directed against *C. albicans* in the commercial preparation Privigen can directly prevent fungal-mediated epithelial damage resembling neutralizing activity. Moreover, we found that *C. albicans* Saps can cleave human antibodies but the cleavage does not interfere with the protective capacity of these antibodies. Our findings reveal a previously undisclosed protective capacity of the human anti-*Candida* antibody response and supports the potential of passive or active vaccination approaches for protection of at-risk patients from invasive fungal disease.

The presence of anti-*Candida* antibodies in Privigen, a pharmaceutical IgG preparation from pooled donor serum, is not surprising as such. As many as 70% of the human population are colonized with the fungus, and *C. albicans* intestinal colonization has been shown to increase specific serum IgG responses [8,25]. Moreover, human serum has been shown to contain a substantial amount of antibodies directed against conserved fungal cell wall components such as mannans or glycosylated proteins [27–29], which will also react to *C. albicans*. This is different to laboratory SPF mice that are usually not colonized with *C. albicans* and whose serum therefore is not expected to contain significant amounts of antibodies binding to *C. albicans*. The lack of specific antibodies in mice may thus explain early studies showing no effect of B cell deficiency in acute murine models of systemic candidiasis [30,31]. Indeed, a recent *in vivo* study using mice colonized with *C. albicans* highlighted both the role of colonization for antibody induction, and the protective role of B cells and antibodies in colonized mice [8].

Our study highlights the protective potential of human antibodies. Naturally occurring human IgG antibodies contained in Privigen or human serum reduce adherence of *C. albicans* to epithelial and endothelial cells and interfere with tissue invasion and destruction of epithelial cells. Accordingly, it can be assumed that the destructive capacity of *C. albicans* cells invading tissues surrounding sites of mucosal colonization will be reduced by the human serum antibody response. This can occur independently of immune cells by a mechanism resembling neutralization, as shown here. *In vivo*, opsonization additionally supports protection by increasing pathogen recognition by phagocytic cells [32,33]. Evidently, the immunological potential of an anti-*Candida* antibody response will, under normal circumstances, be redundant with the capacity of other immune components (especially neutrophils as main effector cells of the innate immune system [34,35]) to clear the fungus, explaining the lack of enhanced *C. albicans* susceptibility in patients lacking B cells or antibodies [36,37].

However, under conditions of immunosuppression, i.e. failure of certain cellular components of the immune system that clear the fungus in immunocompetent settings, the protective power of a human anti-*Candida* antibody response may become of particular importance, and offers a chance for preventive measures in at risk patients. Evidently, for such approaches it would be highly desirable to know target structures of the protective antibodies. We detected interference with adherence and invasion by induced endocytosis, implying that naturally occurring antibodies target surface structures that may serve as adhesins or may be recognized by receptors mediating fungal endocytosis by host cells, such as Als3, or that mediate adherence in another way. Our experiments with mutant cells lacking known adhesins suggest, however, that the complexity of the human response goes beyond single targets. In fact, all of the inhibitory effects of human antibodies that we observe may be mediated by their binding to more than one target, which will hamper their identification, but at the same time boost robustness of the protective response.

Antibodies are frequently cleaved by microbial pathogens, which is considered a mechanism of immune evasion [21]. Cleavage of antibodies by *C. albicans* culture supernatant was also observed [21,23], implying that the occurrence of this mechanism extends beyond the bacterial kingdom. We show here that secreted aspartic proteases, more specifically members of the Sap1-3 subfamily, but not members of other subfamilies such as Sap4-6, are capable of this cleavage. Since only the Fab or F(ab)_2_ fragment appears to remain intact, similarly to pepsin cleavage, antibody functions requiring the effector domain might be impaired, such as complement activation or opsonization for improved phagocytosis of the pathogen. However, we show that the protective capacity mediated by the Fab or F(ab)_2_ fragments remains intact, as we found that they are able to inhibit *C. albicans* adherence.

It should be noted that antibody cleavage by *C. albicans* Saps in an *in vivo* setting has not been shown so far. In fact, the conditions we used to detect cleavage would rarely be matched under physiological circumstances. Considering this, and the fact that major protective capacities of the antibodies remain intact, one may conclude that a previously recognized potential mechanism of immune evasion of *C. albicans* from antibody responses is not very potent, raising the awareness of the robustness of the human anti-*Candida* antibody response. We would, therefore, like to stress the potential of antibodies for treatment options via passive or active immunization of at-risk patients, encouraging more research on their target structures and optimal vaccination approaches, and raising consideration of the B cell response to *C. albicans* as potentially protective.

## Material and methods

### Candida strains and cell lines

In this study, the *C. albicans* strains SC5314, M1285 (ALS3 wildtype), M1284 (*als3Δ*), M1477 (*als3Δ +ALS3*), M35 (SSA1 wildtype), M2068 (*ssa1Δ*) and M2342 (*ssa1Δ+SSA1*) were used [38]. *C. albicans* cultures used for adherence, invasion or damage assays were prepared by inoculating a single colony in YPD (1% yeast extract, 2% glucose, 2% peptone) medium and incubation over night at 30°C and 180 rpm. In order to generate heat killed *C. albicans* cells (SC5314), an overnight culture was grown in YPD medium at 30°C and 180 rpm. The fungal cells were washed three times in PBS. A subsequent overnight culture in YNB (0,67% yeast nitrogen base, 1% glucose) medium was inoculated with 1 × 10^6^ cells/mL and grown at 30°C 180 rpm. After washing two times in PBS, the yeast cells were killed by incubation at 80°C for 15 min.

THP-1 cells used in endocytosis assays were cultured in RPMI1640 medium (Thermo Scientific) complemented with 10% FCS (Sigma Aldrich), 100 µg/µL Penicillin/Streptomycin (Gibco), 2 mM glutamine (Gibco) and 1 mM sodium pyruvate (Gibco). Passages 4 to 24 were used for experiments.

HUVEC cells used in adherence assays were kindly provided by Regine Heller’s group (Universitätsklinikum Jena). Prior to the experiments, cells were isolated from umbilical cords and grown in M199 growth medium (Lonza, Switzerland) containing 15% FCS (Sigma Aldrich), 5% human serum (Sigma Aldrich, Germany), and 7.5 μg/ml endothelial mitogen (Sigma Aldrich, Germany) on 12 mm glass cover slips coated with 1% gelatin until 100% confluence. For adherence, invasion and damage assays, TR-146 cells (passages 4 to 16) cultured in DMEM (Dulbecco’s Modified Eagle’s Medium, Invitrogen) with 10% FCS (Sigma Aldrich) on 12 mm glass cover-slips were used at 100% confluence.

All human cells were maintained at 37°C and 5% CO_2_.

### Monocyte interaction assays

THP-1 cells were labeled for flow cytometry by incubation with 1 µM CFSE (Vybrant CFDA SE Cell Tracer Kit, Thermo Fischer Scientific) for 10 min at 37°C followed by 20 min of incubation in RPMI medium at 37°C. 1 × 10^6^ heat-killed *Candida* yeast were stained for flow cytometry using Calcofluor White (CFW, 1 mg/mL, Sigma Aldrich), washed three times with PBS and incubated with 1.5 µL (Figure 1 A), 0.1 µL, 0.5 µL, or 2.5 µL (Figure 1 B) of commercially available human serum pooled from healthy donors (Biochrom AG/Merck Millipore), antibody-depleted serum (BBI Solution), or the human IgG preparation Privigen (CSL Behring) in PBS for 30 min in 37°C with 650 rpm agitation. The yeast cells were washed three times with PBS. For the monocyte interaction assay, 1 × 10^6^ CFW stained yeasts and 5 × 10^5^ CFSE stained THP-1 cells were co-incubated for 1 h at 37°C in 5% CO_2_. The cell suspensions were analyzed using a LSR Fortessa cytometer (BD Biosciences).

### Adherence and invasion assays

The adherence and invasion assays were performed as described by Wächtler *et al*. 2011 [17]. Briefly, the cell culture medium of the respective human cells was replaced by serum-free cell culture medium supplemented with human serum (Biochrom AG/Merck Millipore), Privigen (CSL Behring), IgA (Sigma-Aldrich) or anti-fungal β-glucan (Thermo Fisher) and *C. albicans* yeasts at a final concentration of 1 × 10^5^ *C. albicans* cells per mL. In some invasion assay samples (Figure 3), the TR-146 cells were incubated with 2.5 µM/mL Cytochalasin D (Sigma Aldrich) for 45 min at 37°C and 5% CO_2_ before the addition of *C. albicans* cells (living or killed by 30 min exposure to UV light (0.12 mJ/cm^2^).

In order to determine the adherence of *C. albicans* yeasts, fungal and human cells were co-incubated for 30 min or 1 h at 37°C in 5% CO_2_, internalization/invasion assays were co-incubated for 3 h at 37°C in 5% CO_2_ and damage was assessed after 24 h incubation at 37°C.

Non-adherent fungal cells were removed by washing and samples were fixed with Histofix (Carl Roth). After blocking of unspecific binding sites with 5% BSA, *C. albicans* cells adherent to the outside of human cells were stained with rabbit anti-*Candida* (2 μg/mL, Acris Antibodies) and anti-rabbit IgG-AF488 (0.4 μg/mL, Thermo Scientific). For invasion assays, TR-146 cells were permeabilized with 0.5% Triton X-100 (Sigma Aldrich) and stained with Dylight Phalloidin-AlexaFluor 594 (2.5 Units/mL, Thermo Scientific). Internalized parts of the *C. albicans* cells were marked using rabbit anti-*Candida* antibodies (2 μg/mL, Acris Antibodies) and anti-rabbit IgG-AF647 (0.4 μg/mL, Thermo Scientific).

An Axio Observer.Z1 fluorescence microscope (Zeiss) was used to take pictures of the cells growing on coverslips with ten-fold magnification. Adherence assays were evaluated by counting the *C. albicans* yeasts on the fully confluent TR-146 or HUVEC cell layer on 10 pictures that were taken throughout each coverslip. The invasion of *C. albicans* was analyzed by counting the hyphae with internalized portions among at least 150 hyphae on each coverslip.

### Damage assays

To assess epithelial cell damage, confluent TR-146 cells were co-incubated with 2 × 10^4^ *C. albicans* cells/ml in DMEM supplemented with human serum or Privigen for 24 h. As a control for high cellular damage, some TR-146 cells were treated with 5% Triton X-100 (Sigma Aldrich). Culture supernatants were collected and tested for released lactate dehydrogenase (LDH) activity using the Applied Science Cytotoxicity Detection Kit (Roche). The OD of the samples was measured with the Infinite M200 pro reader (Tecan) at 490 nm. Background signals measured at 600 nm were subtracted before analysis.

### Cleavage of antibodies by Saps and pepsin

Recombinant Sap proteinases were expressed in *Escherichia coli* (Sap2) or *Pichia pastoris* and purified as previously described [39]. 100 µg (Figure 5(a) or 7.5 µg Privigen, IgG (Jackson ImmunoResearch), IgA (Sigma Aldrich) or IgM (Jackson ImmunoResearch) were incubated with different amounts of *C. albicans* Saps or pepsin (Carl Roth) for 1 h (Figure 5(a-c)) or overnight (Figure 5(d-e)) at 37°C in citrate buffer with either pH 5.2, or the pH optimum matching the respective proteinase (pH 3.2 for pepsin, Sap1, Sap2 and Sap3; pH 5.2 for Sap5 and Sap6; pH6.5 for Sap9 and Sap10).

Samples containing 15 µg (Figure 5(a-c)) or 50 µg (Figure S3 and Figure 5(e)) Privigen were prepared for SDS-PAGE (12% polyacrylamide gels) in Laemmli buffer by incubation at 95°C for 5 min and addition of 2 µL 1 mM DTT. Gels were either stained with Coomassie Quick stain solution (Serva) overnight or used for Western blotting procedures. Western blotting was performed using a wet transfer system. Prior to detection, nitrocellulose membranes were blocked with PBS containing 5% milk powder for 1 h. Incubation with the primary antibody anti-human IgG-biotin (1 µg/mL, BD BioScience) diluted in PBS with 5% milk powder was performed overnight. Nitrocellulose membranes were washed with PBS and incubated with streptavidin conjugated to horse radish peroxidase (Biolegend) diluted in PBS with 5% milk powder for further 3 h. Western blots were evaluated by exposure of X-ray films (Christiansen & Linhardt) to the membranes submerged in ECL (GE Healthcare).

### Mass spectrometry & protein database search

The mass spectrometry proteomics data have been deposited to the ProteomeXchange Consortium via the PRIDE [40] partner repository with the dataset identifier PXD026082. The LC-MS/MS analysis method and the protein database search approach were described in detail in the sample processing protocol and the data processing protocol of the PRIDE dataset (https://www.ebi.ac.uk/pride/archive/projects/PXD026082).

### Data analysis

Flow cytometry data were analyzed using FACSDiva and FlowJo V. 10.1 (BD Bioscience). Fluorescence microscopy pictures were evaluated with Zen 2 Blue edition (Zeiss) and ImageJ (NIH). Statistically significant differences between samples were determined by a calculation of the variance using an F-test, followed by unpaired two-tailed student’s t-test for equal or unequal variances, respectively. Statistical analysis was performed using Microsoft Excel Software. p values lower than 0.05 were considered significant (*p < 0.05, **p < 0.01, ***p < 0.001).

## Supplementary Material

Supplemental Material

## Data Availability

All data and materials that support the results or analyses presented in this paper are freely available under https://github.com/bjungnickel/wich.
