# Supplementary figures and images for: Functionality of the human antibody response to Candida albicans
Source: Virulence. 2021 Dec 19;12(1):3137–48. doi: 10.1080/21505594.2021.2015116 (PMC8923069; doi:10.1080/21505594.2021.2015116)

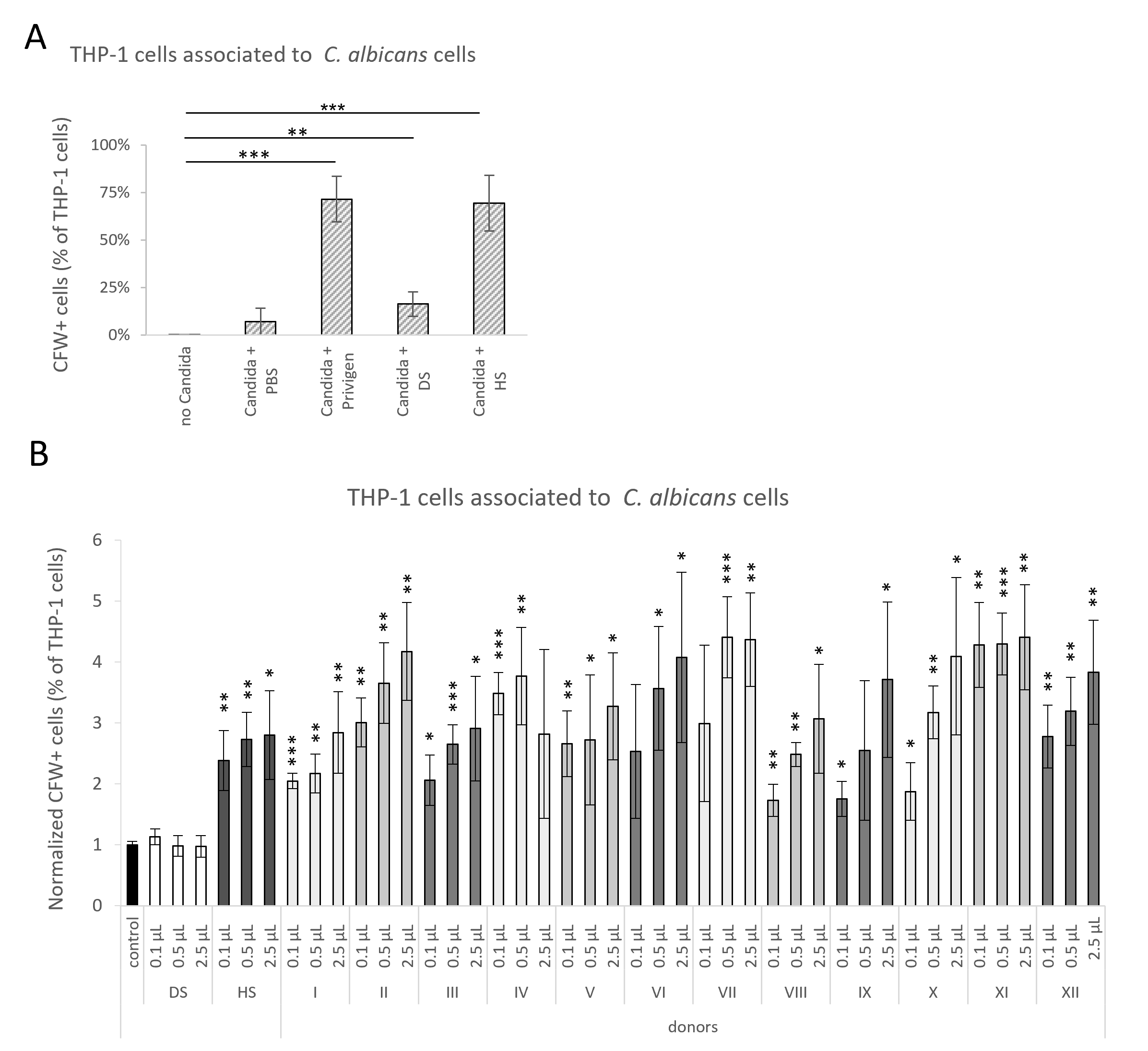

Supplement: Supplemental Material [file KVIR_A_2015116_SM5108.zip › supplementary/Supplementary_Figure_1.png]

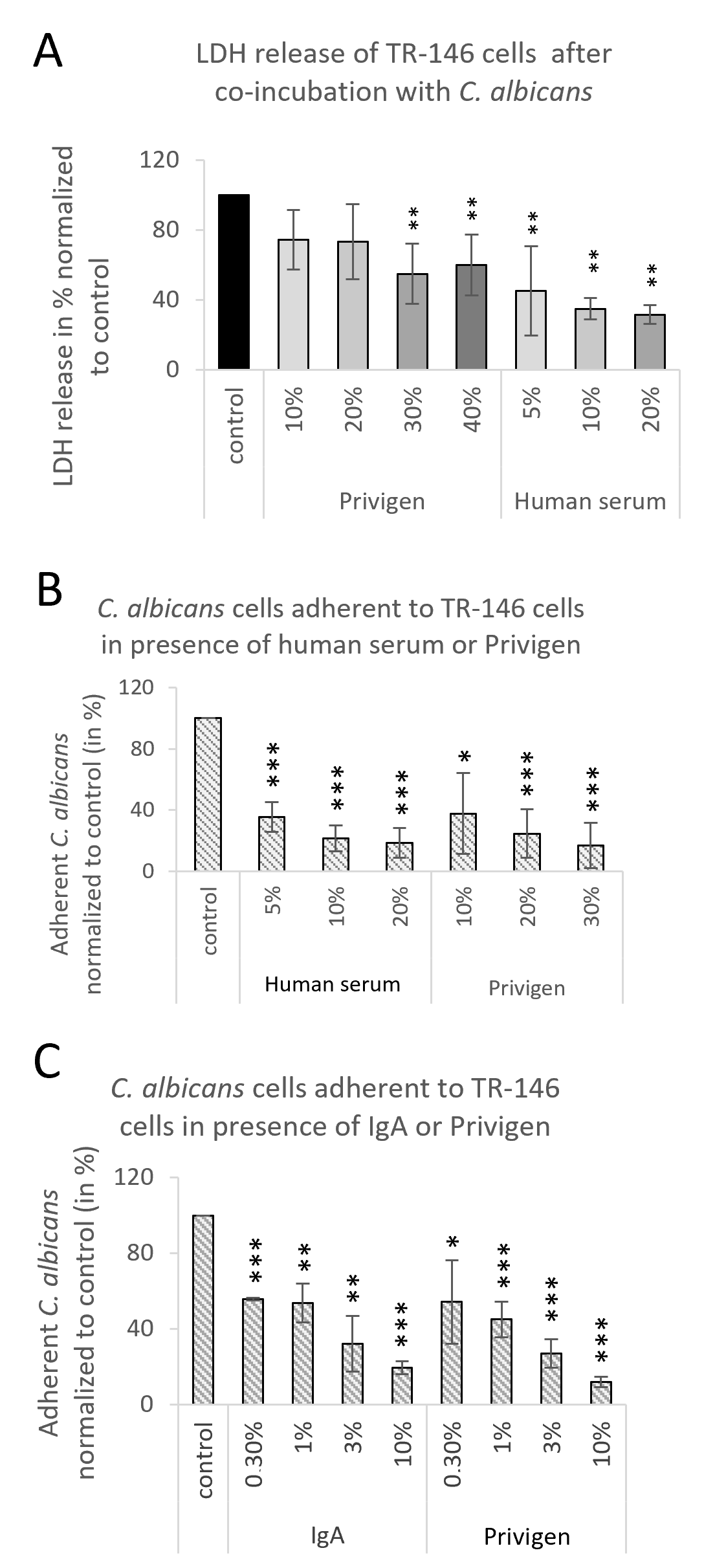

Supplement: Supplemental Material [file KVIR_A_2015116_SM5108.zip › supplementary/Supplementary_Figure_2.png]

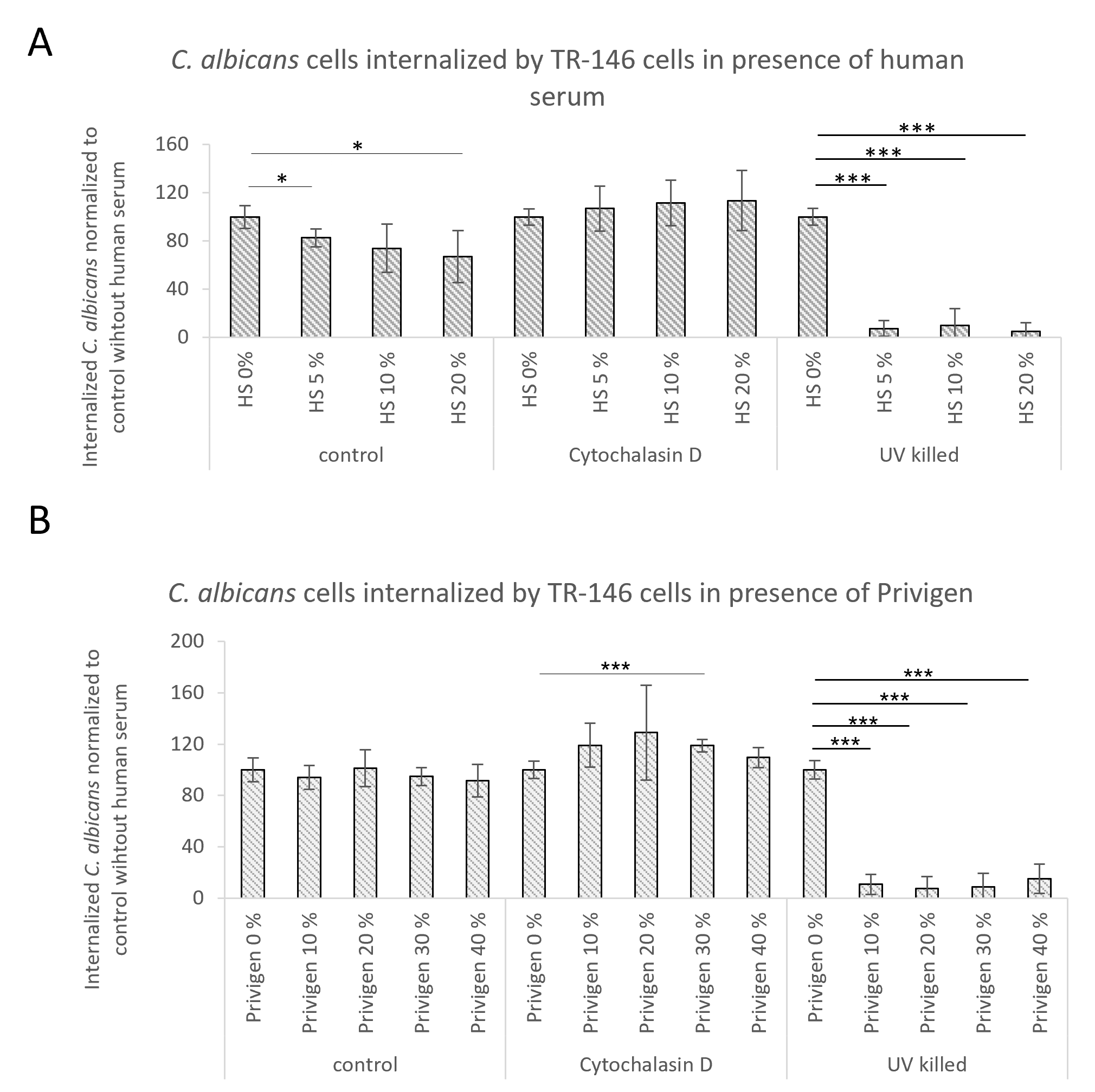

Supplement: Supplemental Material [file KVIR_A_2015116_SM5108.zip › supplementary/Supplementary_Figure_3.png]
